# Supplementary figures and images for: Genome-wide analysis of cellulose synthase (CesA) and cellulose synthase-like (Csl) proteins in Cannabis sativa L
Source: PeerJ. 2024 Jul 31;12:e17821. doi: 10.7717/peerj.17821 (PMC11636989; doi:10.7717/peerj.17821)

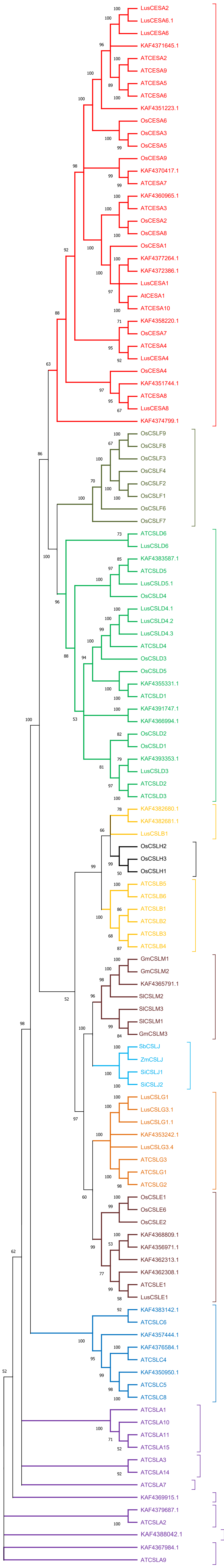

Supplement: Figure S1 [file peerj-12-17821-s001.pdf]

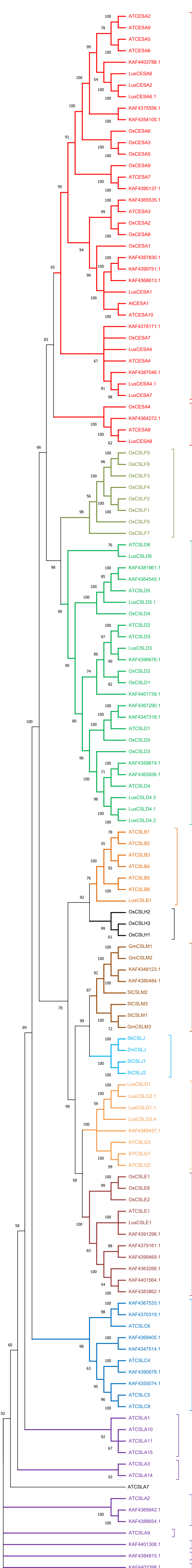

Supplement: Figure S2 [file peerj-12-17821-s002.pdf]
